# Toward an epistemic grounding of clinical reasoning: artificial intelligence and psychological diagnosis

**DOI:** 10.3389/fdgth.2026.1952196

**Published:** 2026-10-07

**Authors:** Andrea Primo Pierotti, Marco Cremaschi

**Affiliations:** 1 Mental Health Department, Niguarda Hospital, Milan, Italy; 2 Department of Informatics, Systems and Communication - DISCo, University of Milano-Bicocca, Milan, Italy; 3 Whattadata S.r.l., Milan, Italy

**Keywords:** abductive reasoning, artificial intelligence, clinical reasoning, epistemology, psychological assessment, psychological diagnosis, psychopathology

## Abstract

Artificial intelligence (AI) is increasingly used to support diagnosis across medicine, yet extending its success to psychological assessment raises a distinct epistemological problem. Psychological diagnosis requires interpreting subjective experience, contextualizing clinical information, and developing explanatory hypotheses within the clinician-patient relationship. In this Perspective, we compare the inferential structure of medical and psychological diagnosis and argue that psychopathological reasoning relies especially on abduction, namely the generation and iterative revision of plausible explanations when the available evidence underdetermines a single account. Our conceptual contribution is to propose that the intersubjective clinical encounter, including the clinician’s affective and experiential resonance, may provide an important grounding condition for this abductive process. Current AI systems can support induction, deduction, classification, evidence retrieval, consistency, and the exploration of alternative hypotheses. However, even when conversational or multimodal, these systems process representations of the clinical encounter; such interaction does not ensure experiential and intersubjective participation in the interpersonal processes through which clinically meaningful information is generated. Given these epistemic uncertainties, their outputs should not be treated as substitutes for psychological formulation or as epistemically privileged representations of patients. We propose three principles for responsible integration: characterize systems according to their actual epistemic operation; adopt epistemic humility by treating algorithmic outputs as provisional hypotheses; and pursue complementarity rather than substitution. AI should therefore be evaluated and deployed according to the epistemic operation it performs, and to the specific component of diagnostic reasoning it supports, within a multimethod assessment framework.

## 1 Introduction

Artificial intelligence (AI) has demonstrated remarkable progress across several areas of medicine, where it has become increasingly integrated into clinical decision-making and diagnostic workflows. Applications now span several clinical tasks, including image-based detection and diagnosis across radiology, ophthalmology, digital pathology, and endoscopy (1–3); automated interpretation of physiological signals (4, 5); risk prediction from longitudinal electronic health records (6); and real-time patient monitoring and early-warning systems (7), collectively supporting broader clinical decision-making (8).

Although these applications address heterogeneous clinical problems, they share an important epistemic feature: diagnostic decisions are typically formulated as the identification or prediction of relatively well-defined clinical states from standardized sources of information. This makes it possible to evaluate AI systems against shared reference standards and measurable clinical outcomes.

Whether these assumptions can be directly extended to psychological diagnosis, however, remains an open question. In this Perspective, we examine psychological diagnosis from an epistemological perspective, informed by phenomenological and psychodynamic approaches, using medical diagnosis as a point of comparison rather than contrast. We argue that some of the abductive components of psychopathological diagnostic reasoning are grounded in the intersubjective clinical encounter and in the clinician’s experiential resonance, and we consider how this specificity both delimits and guides the role of AI in psychological assessment.

## 2 The clinical encounter as a hermeneutic and relational process

In medical interviewing, physicians are informed of the patient’s presenting complaint and seek to identify its etiology and pathogenesis. The patient’s symptoms constitute the primary explanandum, while diagnosis aims to identify the disease process responsible by relating them to the underlying biological mechanisms (9, 10).

In psychological interviews, clinicians are rarely confronted with a direct path from the patient’s presenting complaint to the underlying source of psychological suffering. They face subjective experiences that are not directly accessible to observation and must be progressively interpreted through the patient’s narrative (11), which relies on the patient’s capacity to understand and articulate their own inner experience. This narrative is seldom fully transparent: individuals may only partially recognize the origins of their distress, struggle to identify what is clinically relevant, or be reluctant to disclose personally meaningful experiences because of shame, defensiveness, or ambivalence toward change (10).

This process is inherently hermeneutic, as assessing a mental state involves two successive interpretative reductions. The first occurs when the patient attempts to translate an internal experience into language; the second occurs when the clinician seeks to understand the meaning of that account. This process culminates in a hermeneutic synthesis integrating the patient’s narrative with the clinician’s interpretative framework into a clinically meaningful formulation (11, 12).

Rather than simply eliciting information, the clinical encounter creates the conditions for generating meaningful questions, exploring recurrent themes, and developing a shared understanding of the patient’s difficulties (13). This exploratory process necessarily unfolds over time, as the initial themes brought by the patient are progressively refined, revised, and sometimes fundamentally transformed through the ongoing dialogue between clinician and patient (10).

Psychological assessment, understood as the process of acquiring and integrating information, typically relies on multiple sources, including self-report questionnaires, performance-based tests, and behavioral measures (14, 15). Within this process, clinical reasoning supports psychological diagnosis, understood not as the mere assignment of a diagnostic label but as the development of an explanatory understanding of the individual’s psychological functioning. The focus is not only illness-oriented but also person-oriented.

The diagnostic process should not be considered complete once sufficient symptoms have been identified to satisfy a descriptive diagnostic category, as those listed in traditional psychiatric nosographies. In clinical psychology, the limitations of purely descriptive diagnostic systems (such as the Diagnostic and Statistical Manual of Mental Disorders) have long been recognized. These systems primarily provide a nomothetic framework for understanding mental disorders, identifying symptoms and characteristics that individuals share with others with the same disorder, but offer limited understanding of the implicated etiological mechanisms (16). This perspective benefits from a complementary idiographic understanding of the individual, in which symptoms acquire clinical meaning only within the broader context of the person’s personality functioning, life history, and interpersonal relationships (17). Stopping the assessment prematurely risks reducing diagnosis to symptom classification rather than a case formulation, namely an individualized explanatory account of how those symptoms emerge and are maintained (10, 18).

The different sources of information in psychological assessment contribute to a clinically meaningful understanding of the patient’s experience, upon which explanatory hypotheses can subsequently be formulated.

## 3 Abductive clinical reasoning

The exploratory and interactive nature of psychological assessment has important implications for the type of reasoning involved in psychological diagnosis. Although clinical reasoning is sometimes portrayed as a hypothetical-deductive process in which symptoms are matched to diagnostic categories, both medicine and psychology rely on a more complex inferential structure in which abduction plays an important role. This inferential architecture has been most extensively described in the medical literature, where diagnosis typically begins with abduction, through which clinicians generate plausible explanations for the available clinical information. These hypotheses are then subjected to deduction, whereby clinicians derive the clinical findings expected if a given hypothesis were correct, guiding further examination and testing. Finally, induction evaluates those expectations against newly acquired evidence, allowing hypotheses to be supported, revised, or discarded and, where necessary, initiating further cycles of abductive hypothesis generation and testing (19).

The hypotheses generated through this process are continuously revised as new observations become available, and judged by their capacity to account coherently for the evolving clinical picture (20–22). Clinical abductive reasoning, however, is not a matter of arbitrary intuition, but of the creative generation of hypotheses that is constrained by clinical phenomena, the relational encounter, theoretical knowledge, and criteria of explanatory coherence. Accordingly, abduction is best understood as an epistemically disciplined form of creativity rather than an unconstrained associative process (22).

The relative weight of these inferential processes differs substantially between medicine and psychopathology. In some areas of medicine, the diagnostic task is oriented toward identifying diseases whose underlying biological mechanisms are, at least in principle, sufficiently established to allow hypotheses to be progressively corroborated or rejected through objective findings. Although uncertainty remains an intrinsic component of medical practice, in many cases the inferential process is progressively constrained by biological evidence and standardized diagnostic procedures.

In psychological assessment, the mechanisms underlying a patient’s symptoms and subjective experiences are neither directly observable nor directly inferable from diagnostic categories. Rather, they must be inferred through a process that progressively develops explanatory hypotheses about the individual’s psychological functioning (21). This process extends beyond the identification of symptoms to explain how seemingly disparate observations, including symptoms, behaviors, verbal expressions, psychometric findings, interpersonal patterns, and developmental experiences, can be integrated into a coherent and sophisticated account of the person’s difficulties (15). Crucially, as introduced in Section 2, these observations are not self-interpreting. Their clinical significance depends on the meanings they acquire within the patient’s broader psychological organization, so that identical verbal expressions or behaviors may reflect profoundly different underlying processes across individuals.

The difference between medicine and psychology is therefore not that one relies on abductive reasoning whereas the other relies on deduction, but that psychological diagnosis depends much more extensively on abductive inference because its explanatory target is neither directly observable nor exhaustively specified by diagnostic categories alone.

When clinical presentations are particularly complex, the creative dimension of abduction becomes especially important, as it permits the generation of a new explanatory rule that makes sense of the observed phenomena. In these situations, abduction does not simply select among existing explanations but proposes a novel explanatory hypothesis for a singular clinical presentation.

More generally, abductive reasoning becomes indispensable whenever the available evidence underdetermines a single explanation, that is, when the same observations can reasonably support multiple competing hypotheses. Underdetermination also occurs in medicine, and it is particularly salient in rare, atypical, or previously unknown conditions, where established explanatory models prove insufficient. In psychological assessment, however, underdetermination is not an exception but an intrinsic feature of diagnostic reasoning. Clinical presentations frequently involve overlapping syndromes, heterogeneous symptom configurations, ambiguous narratives, and contextual factors that resist straightforward classification within existing diagnostic categories (20, 23).

This raises a fundamental question: what constrains and guides the generation and progressive refinement of explanatory hypotheses in psychological diagnosis?

## 4 The clinical encounter as a possible grounding for abduction

Acknowledging the hermeneutic nature of the psychiatric and psychological clinical encounter, the clinician’s understanding of the patient’s subjective experience necessarily involves an empathic mode of listening through which the clinician attempts to grasp “what it is like” to experience a particular symptom or to live with a given mental disorder (11).

In this sense, the hermeneutics of the clinical encounter concerns the progressive understanding of how a particular phenomenon is experienced within the patient’s field of consciousness. This process necessarily engages the clinician’s own subjectivity. Although subjectivity may introduce biases into clinical reasoning, it also constitutes an essential instrument of understanding. The clinician’s emotional responses, intuitive impressions, and experiential resonances become clinically meaningful sources of information, many of which remain implicit and difficult to articulate explicitly (10, 11, 24). The clinical encounter has also been discussed as involving important creative components, based on the clinician’s capacity for associative fantasizing and generating lateral associations between the patient’s lived experience and their own experiential repertoire (25).

As an inherently interpersonal process, this form of understanding is shaped by embodied, relational, and neurobiological processes. Different theoretical perspectives have emphasized complementary aspects of these processes: Schore has discussed affective attunement and interpersonal synchronization (26), while Fuchs has highlighted the embodied and relational dimensions of human understanding (27), Gallagher has conceptualized interpersonal understanding in terms of embodied and situated interaction (28), and De Jaegher and Di Paolo have discussed how meaning can emerge and transform through interpersonal interaction (29).

Building on these observations, we propose that this process offers one possible epistemic grounding for abductive reasoning, allowing clinicians to anchor the creative generation of explanatory hypotheses in their experiential engagement with the patient’s lived experience. This perspective has important implications for the extent to which AI can support clinical reasoning in this domain.

Recent advances in AI have demonstrated remarkable performance in the inductive and deductive components of clinical reasoning. Current systems are increasingly capable of extracting statistical regularities from complex datasets, reducing noise, identifying inconsistencies, and supporting hypothesis verification within diagnostic workflows (20, 30).

By contrast, the ability of AI systems to engage in abductive reasoning remains a matter of ongoing debate and appears to depend on the particular form of abduction under consideration. The recent AI-generated disproof of the Erdős unit distance conjecture provides compelling evidence that large language models can indeed exhibit a specific and generative form of abductive reasoning. However, as Gowers observes (31), the epistemic demands of constructing a counterexample may differ from those required to produce genuinely novel theoretical frameworks, suggesting that current AI systems may excel only for particular classes of problems. We think that Gowers’ observation is significant because it highlights that the Erdős result is not, by itself, sufficient to unequivocally establish that AI can perform the kind of creative abductive synthesis involved in clinical reasoning.

A suggestive perspective in this regard is offered by Zahavy (30). Drawing on Einstein’s reflections on scientific discovery, Zahavy argues that the creative abductive leaps require being grounded in embodied interaction with the physical world. Einstein’s formulation of the equivalence principle illustrates this point: rather than emerging from the accumulation of new observations or from the logical application of existing theories, it originated from a thought experiment grounded in the imagined experience of a freely falling observer, from which a novel explanatory hypothesis about gravity emerged.

Zahavy proposes that future AI systems may progressively acquire interactive world models operating on coherent latent representations of physical reality, and may eventually approximate forms of abductive reasoning grounded in increasingly sophisticated models of the physical world. We think that psychological diagnosis, however, presents a qualitatively different epistemic challenge. We believe that its grounding does not arise primarily from interaction with external reality, but from the evolving interpersonal encounter through which meaning itself is co-constructed between clinician and patient.

If this is the case, reproducing this specific form of creative abductive synthesis may depend on conditions whose precise nature remains difficult to specify. We do not yet fully understand what epistemic implications may follow from the differences between the architectures and forms of information processing underlying human and artificial systems, nor do we have established criteria for determining when an AI system might participate in an intersubjective encounter in a manner comparable to human clinicians. At present, we do not have sufficient grounds to treat AI-generated clinical reasoning as epistemically equivalent to that of a human clinician.

To date, we do not yet have an established account of what would demonstrate experiential or intersubjective participation in an artificial system, and behavioral performance alone would not establish this. Nevertheless, the proposal remains open to empirical challenge. For example, it would be weakened if controlled studies showed that AI systems could generate and iteratively revise patient-specific clinical formulations with comparable quality across diverse cases when provided only with information made available through the interaction, without access to the additional relational and experiential information available to clinicians. The same question would arise if AI systems participating directly in clinical encounters achieved comparable results, even without independent grounds for determining whether such interaction involved experiential or intersubjective participation. Neither scenario would by itself establish the presence or absence of subjective experience in AI systems, but either would weaken the claim that experiential and intersubjective participation constitutes an important epistemic condition for the form of clinical abductive reasoning.

Clinical supervision provides a further boundary case, as supervisors may contribute to clinical formulation without having directly encountered the patient.

## 5 Discussion

Psychological assessment is inherently a multimethod enterprise, in which clinical understanding emerges through integrating multiple sources of evidence rather than from any single instrument or observation. Within this framework, clinicians’ subjective engagement during the clinical interview represents one epistemic component among several. Rather than replacing this component, AI should be integrated according to the specific epistemic contribution it can provide within the broader assessment process.

Recent work has documented rapidly expanding applications of AI and LLMs in mental health, while also highlighting challenges concerning clinical validation, reliability, transparency, and safe integration into clinical practice (32, 33). We propose three recommendations for the responsible integration of AI into clinical reasoning.

### 5.1 Epistemic characterization

First, AI systems should be characterized according to the epistemic operations they actually perform rather than the clinical tasks they appear to accomplish.

The epistemic role of such processing depends on the clinical task it supports. When the task is diagnostic classification, for example, they identify statistical regularities within codified symptoms, traits, or behavioral features and assign diagnostic labels according to predefined criteria. In this case, their function is primarily classificatory: they organize and classify representations of the clinical encounter. Such systems should therefore be understood as supporting the descriptive, rule-based components of diagnostic reasoning, and their performance can appropriately be evaluated using metrics such as diagnostic accuracy, sensitivity, and specificity.

A different epistemic claim arises when AI systems are presented as generating or revising diagnostic hypotheses, or approximating clinical reasoning itself. Here, the relevant operation is no longer classification but abduction: generating explanatory hypotheses that account for the patient’s presentation. Although recent models may assist clinicians by suggesting plausible hypotheses or highlighting overlooked associations, they should not automatically be equated with human abductive reasoning. The distinction is particularly important because, in our view, abductive reasoning in psychological assessment may be grounded not only in the information available to the reasoner but also in the subjective, relational, and experiential processes through which clinically meaningful information emerges.

While contemporary AI systems increasingly include conversational and multimodal models capable of interacting with patients and clinicians and processing heterogeneous clinical information (33), we currently have insufficient grounds to believe that conversational or multimodal interaction itself establishes experiential and intersubjective participation in the clinical encounter.

### 5.2 Epistemic humility

Second, the integration of AI into clinical reasoning requires epistemic humility. It has been argued that AI should not be granted epistemic privilege over patients’ subjective testimonies or clinical dialogue (34). We agree with this position but suggest that epistemic humility has broader implications for AI-assisted psychological assessment.

From this perspective, epistemic humility also means recognizing that AI systems operate within a form of reasoning that captures only part of the epistemic work involved in psychological diagnosis. Rather than being treated as definitive representations of a patient’s psychological condition, AI-generated outputs should be regarded as provisional hypotheses to be explored, tested, and refined through an iterative diagnostic process. The principal risk, therefore, lies in confusing computational confidence with epistemic or ontological certainty.

This distinction is particularly important given the epistemic status of psychiatric diagnoses themselves. Unlike many areas of medicine, psychiatric diagnoses are not universally regarded as natural entities corresponding to discrete biological conditions, but are increasingly understood as provisional explanatory models rather than as natural kinds with fixed essences (35). Consequently, the apparent precision and objectivity of algorithmic predictions, particularly when expressed as diagnostic labels, should not encourage clinicians to treat diagnostic classifications as privileged representations of psychological reality.

Recent work on AI-assisted clinical decision-making further highlights the importance of calibrated uncertainty and appropriate reliance on AI outputs. Explanations do not necessarily calibrate clinicians’ trust or protect them from systematically biased recommendations (36, 37), while LLMs’ verbalized confidence may itself be systematically overconfident (38). AI-generated outputs should therefore remain provisional: their uncertainty and evidential limitations should be communicated explicitly, and clinicians should remain attentive to disagreement between AI-generated recommendations and their independent clinical judgment.

This distinction has implications beyond diagnostic classification. Because psychiatric diagnoses influence how individuals understand themselves and are understood by others, assigning unwarranted epistemic authority to AI-generated classifications may also contribute to reinforcing the looping effects through which diagnostic categories shape subsequent experiences, behaviors, and clinical trajectories (39).

### 5.3 Complementarity rather than substitution

AI should be used to complement, rather than replace, clinicians’ subjectivity. AI can support clinical reasoning by reducing unwanted cognitive noise, increasing the consistency of judgments, retrieving relevant evidence, and prompting consideration of alternative diagnostic hypotheses (20).

Table 1 analyzes different clinical tasks in psychological assessment, distinguishing their epistemic functions, the respective roles of clinicians and AI systems, epistemic considerations, and criteria for evaluating AI contributions.

**Table 1:** One possible operationalization of the proposed epistemic division of labor between clinicians and artificial intelligence across core tasks in psychological assessment. For each task, the table outlines its epistemic function, the clinician’s role, the potential contribution of AI, epistemic considerations relevant to its appropriate use, and possible task-specific validation criteria.

Clinical task	Epistemic function	Clinician’s role	AI contribution	Epistemic considerations	Validation criteria
Information gathering during the clinical interview	Exploration and refinement of clinically relevant themes	Provide the relationship as a space for exploration, allowing the patient’s narrative to unfold and focus progressively on clinically relevant material	Support structured interviews and systematic information collection, and assist clinicians in organizing clinically relevant information	Hermeneutic reduction inherent in the clinical encounter; AI may directly interact with the patient, but such interaction does not by itself establish experiential or intersubjective participation	Accuracy and completeness of information collected; preservation of clinically relevant themes
Scoring and summarization of assessment test results	Calculation, organization, and integration of assessment results	Interpret results in the context of the patient’s presentation and integrate them with information from other assessment sources	Automate scoring, organize results across assessment measures, and generate standardized summaries	Avoid conflating standardized test results with their clinical interpretation	Accuracy of scoring; completeness and faithfulness of summaries
Categorical psychological diagnosis	Classification	Integrate diagnostic information and evaluate hypotheses through clinical reasoning grounded in the clinical encounter	Reduce cognitive noise and support diagnostic classification by prompting consideration of alternative hypotheses	Diagnostic categories do not exhaust the understanding of the patient; provisional ontological status of diagnostic categories; hypotheses should remain subject to revision through clinically grounded evaluation	Diagnostic accuracy, sensitivity, and specificity
Case formulation	Abductive synthesis of heterogeneous clinical information	Integrate multiple sources of information and generate, evaluate, and revise explanatory hypotheses also through clinically grounded experience	Generate alternative explanatory hypotheses and support the evaluation of clinical hypotheses	Formulation should remain open to revision; explanatory hypotheses should not be treated as definitive accounts of the patient	Plausibility and diversity of generated hypotheses; usefulness in identifying alternative explanations

The validation criteria are task-specific and reflect different epistemic standards, with objective performance metrics applying primarily to classification tasks, whereas case formulation requires qualitative and clinically grounded evaluation.

## 6 Conclusions

AI is likely to become an increasingly integral component of psychological assessment, just as psychological assessment itself continues to evolve. In this context, the challenge is understanding how the underlying assumptions of AI systems interact with the epistemological diversity that characterizes contemporary clinical psychology. AI systems are not neutral instruments: they inevitably embody specific theories about what constitutes clinically relevant evidence and how diagnostic conclusions should be reached. An uncritical reliance on these embedded models risks privileging forms of knowledge that are more easily codified, quantified, and predicted, while progressively marginalizing those aspects of assessment that emerge through interpretation, contextualization, and the clinician-patient relationship. Such a shift would not merely affect diagnostic decisions; it could gradually reshape what psychologists themselves come to recognize as legitimate clinical knowledge.

Within this evolving landscape, the clinical relationship should not be regarded simply as a context in which assessment takes place, nor as a limitation that future AI systems will eventually overcome. Rather, it can be regarded as one of the primary sources through which psychological knowledge is generated. Preserving this epistemic role is therefore not an argument against technological innovation, but a prerequisite for ensuring that AI contributes to the advancement of psychological assessment without inadvertently narrowing the ways in which psychological phenomena can be understood.
